# The impact of ordinal scales on Gaussian mixture recovery

**DOI:** 10.3758/s13428-022-01883-8

**Published:** 2022-07-13

**Authors:** Jonas M. B. Haslbeck, Jeroen K. Vermunt, Lourens J. Waldorp

**Affiliations:** 1 Psychological Methods Group, University of Amsterdam, Amsterdam, Netherlands; 2 Department of Methodology and Statistics, Tilburg University, Tilburg, Netherlands

**Keywords:** Mixture modeling, Gaussian Mixture Modeling, Ordinal scales, Misspecification, Model selection

## Abstract

Gaussian mixture models (GMMs) are a popular and versatile tool for exploring heterogeneity in multivariate continuous data. Arguably the most popular way to estimate GMMs is via the expectation–maximization (EM) algorithm combined with model selection using the Bayesian information criterion (BIC). If the GMM is correctly specified, this estimation procedure has been demonstrated to have high recovery performance. However, in many situations, the data are not continuous but ordinal, for example when assessing symptom severity in medical data or modeling the responses in a survey. For such situations, it is unknown how well the EM algorithm and the BIC perform in GMM recovery. In the present paper, we investigate this question by simulating data from various GMMs, thresholding them in ordinal categories and evaluating recovery performance. We show that the number of components can be estimated reliably if the number of ordinal categories and the number of variables is high enough. However, the estimates of the parameters of the component models are biased independent of sample size. Finally, we discuss alternative modeling approaches which might be adopted for the situations in which estimating a GMM is not acceptable.

## Introduction

Gaussian mixture models (GMMs) are a popular and versatile tool for exploring heterogeneity in multivariate data across many disciplines (e.g., Frühwirth-Schnatter, 2006; McLachlan et al., 2019). They are able to detect unobserved groups in the data, allow one to study the means and covariances within each group, and assign a probabilistic group membership to each case. Some of the most popular clustering methods are special cases of GMMs. Latent profile analysis (LPA; e.g., Williams & Kibowski, 2016) and latent trait analysis (LTA) (LTA; e.g., Clinton et al., 2004) are special cases where covariance matrices are constrained to be diagonal and hence only the means and variances are modeled; and also the popular k-means algorithm (Hartigan & Wong, 1979) is a special case of GMMs, in which covariances are diagonal, all variances are equal, and the class memberships are hard-thresholded (e.g., Murphy, 2022). GMMs are typically either estimated using a Bayesian approach (e.g., Gibbs sampling) or the expectation–maximization (EM) algorithm together with a model selection strategy such as the Bayesian Information Criterion (BIC; ; Frühwirth-Schnatter, 2006). The latter has been shown to consistently select the correct number of groups (Leroux, 1992; Keribin, 2000), outperformed other criteria in simulation studies (Steele & Raftery, 2010), and is likely the most widely used model selection criteria for GMMs.

However, the recovery of GMMs is only guaranteed if the data-generating mechanism is indeed a GMM, that is, if the GMM is correctly specified. While there are many ways in which the GMM can be misspecified, one type of misspecification that is highly prevalent across disciplines is that the domain of the modeled variables is not continuous but consists of ordinal categories. This is especially the case in the medical, social, and behavioral sciences. For example, medical diseases are often characterized in stages (e.g., *0* to *IV* for breast cancer) and the severity of psychiatric symptoms are often scored in categories such as *not at all*, *several days*, *more than half the days*, and *nearly every day* (e.g., Cameron et al., 2008). Ordinal categories are also the standard response format in surveys, for example the level of education may be ordered from *primary school* to *doctoral degree*. Similarly, opinions or attitudes are typically measured with ordinal variables on a Likert scale ranging, for example, from *strongly agree*, *agree*, *neutral*, *agree* *strongly agree* (Joshi et al., 2015). In many of those applications, it is of interest to detect subgroups and characterize them in terms of their means and (co-)variances. For example, one might be interested in subgroups differing in the means and covariances between symptoms of mental disorders, which could point to different pathological pathways (e.g., Borsboom, 2017; Brusco et al., 2019). While it would in principle be possible to estimate mixtures of models for ordinal data, these methods are not readily available or not feasible for more than a few categories and variables (we will return to those alternatives in the discussion), and therefore GMMs (or LPA/k-means) are typically used in practice. However, it is unclear to what extent estimation methods for GMMs are impacted by observing ordinal instead of continuous data.

In this paper, we use a simulation study to map out to what extent GMM recovery is impacted by observing variables on an ordinal instead of a continuous scale. Specifically, we simulate data from a large range of GMMs, threshold the continuous variables into $\{2,3,\dots ,12\}$ ordinal categories, and try to recover the data-generating GMM with the EM algorithm and the BIC. Our general finding is that if the number of variables is high enough (about *p* > 5), if the variables have five or more ordinal categories, and the sample size is large enough, then the correct number of components *K* can be estimated with high probability. However, the means and covariances in each component are biased, and this bias cannot be reduced by increasing the sample size. We provide detailed results conditional on characteristics of the data-generating GMM and discuss possible strategies for the problematic situations in which GMM recovery is poor.

## Simulation study

The goal of the simulation study is to explore to what extent GMM recovery with the EM algorithm and the BIC is impacted by observing ordinal variables with *c* categories instead of continuous variables. We study the drop in performance as a function of *c* conditional on the number of true components, the separation between them, the number of variables, and the sample size.

### Setup

#### Considered simulation conditions

**Fig. 1:**
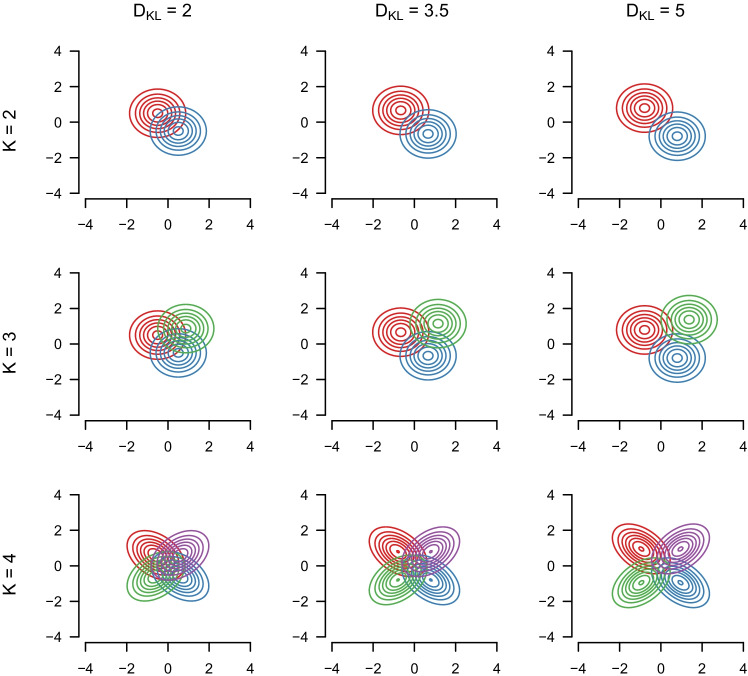
Contour plots of the Gaussian mixture models (GMMs) across variations of the number of components *K* and the pairwise D_KL_ displayed for the *p* = 2 bivariate case

We independently vary the number of components in the mixture models $K\ \in \{2,3,4 \}$, the number of dimensions $p \in \{2, 3, \dots , 10 \}$, the pairwise Kullback–Leibler divergence D_KL_ $\in \{2,3.5,5 \}$ and the total sample size $N\ \in \{1000,2500,10,000 \}$. We choose the data-generating GMMs such that we consider highly overlapping mixture components (D_KL_ = 2), somewhat overlapping mixture components (D_KL_ = 3.5), and clearly separated mixture components (D_KL_ = 5; see Fig. 1).

We choose GMMs such that the pairwise D_KL_ between components are equal, which is necessary to meaningfully compare results across variations of *K*. In the bivariate case (*p* = 2), we can arrange the means of components in an equidistant way for  $K\ \in \{2,3 \}$ and we therefore only vary the means and not the variances and covariances across mixture components. We fix all variances to $\sigma ^{2}=\sqrt {0.25}$ and set all covariances to zero. For *K* = 4, it is impossible to place the component means equidistant from each other, and we therefore vary the covariances to obtain the same D_KL_ as in  $K\ \in \{2,3 \}$. For dimensions *p* ≥ 3, it is again possible for  $K\ \in \{2,3,4 \}$ to place the means at pairwise equidistant locations and we therefore only vary the means and keep the covariance matrix constant. In these cases, we use numeric optimization to choose arrangements of component means that have the desired pairwise D_KL_. For a detailed description for how we defined the means of the mixture components, see Appendix A. We set the mixing probability of each mixture component to $\frac {1}{K}$.

#### Mapping from continuous to ordinal data

**Fig. 2:**
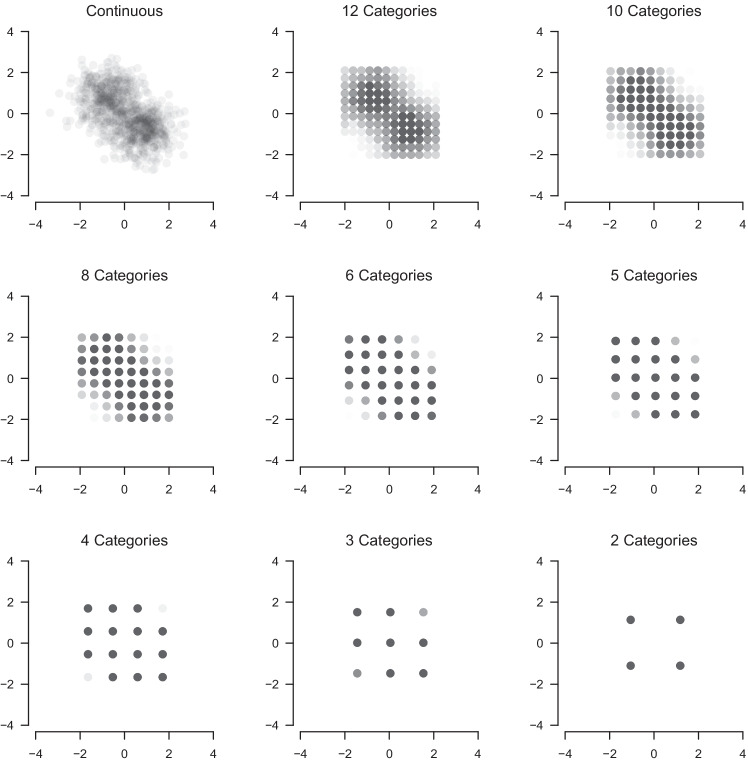
Bivariate Gaussian mixture (*top left*) is mapped to ordinal scales with 12, 10, 8, 6, 5, 4, 3 and 2 categories

Figure 2 illustrates the mapping from continuous variables to ordinal categories with the following procedure. For each variable separately, we calculated the 0.50% and 99.50% quantiles and then construct *c* equally spaced categories between those quantiles. The 1% data lying outside this interval on each side are mapped into the nearest category. We chose this procedure to avoid that extreme values can meaningfully influence the mapping for small sample sizes *N*. This implies that the borders of the grid are defined by the 0.50% and 99.50% quantiles of the Gaussian mixture. The labels of the *c* categories are defined as the midpoints of the intervals defined by the *c* + 1 thresholds (including the boundaries defined by the quantiles as thresholds). For example, if the thresholds are − 1,0,1, then the category labels would be − 0.5,0.5. We choose these category labels to ensure that the ordinal data are on a similar scale as the continuous data. This is required to meaningfully assess estimation errors on the component parameters because it would not make sense to compare the estimated mean of component 1 with corresponding the true mean, if we already know that all values in the observed data are *a* times bigger.

#### Estimation method

We estimated GMMs with the EM algorithm and the BIC using the implementation in the R-package *mclust* version 5.4.6 (Scrucca et al., 2016). To limit the number of parameters of GMMs, we constrained the GMMs to be isomorphic, that is, to have equal variances and covariances. Thus, in this case, the GMM is equal to a latent profile analysis. Note that this class of GMMs is correctly specified for all simulation conditions, except the one for *p* = 2, *K* = 4, where we also varied the covariances. We consider the sequence of $K \in \{1, \dots , 7 \}$. In order to estimate expected performance measures, we compute averages over 100 repetitions of the design. The repetitions use the same mixture models but differ in the generation of Gaussian noise.

In some applications one might be specifically interested in differences in covariances across components. For example, clinical psychologists analyze the covariances between symptoms of mental disorders (Borsboom, 2017) to uncover possible causal interactions between symptoms (however, note that this non-trivial, see e.g., Ryan et al., 2019; Haslbeck et al., 2021). Mixture models have been suggested as a way to determine whether individuals with/without diagnosis differ in their symptom covariances (Haslbeck et al., 2021; Brusco et al., 2019; De Ron et al., 2021). We therefore run the simulation design a second time in which we freely estimate all covariance matrices. The code to reproduce the simulation study and all results and figures in the paper can be found at https://github.com/jmbh/OrdinalGMMSim_reparchive.

### Results

**Fig. 3:**
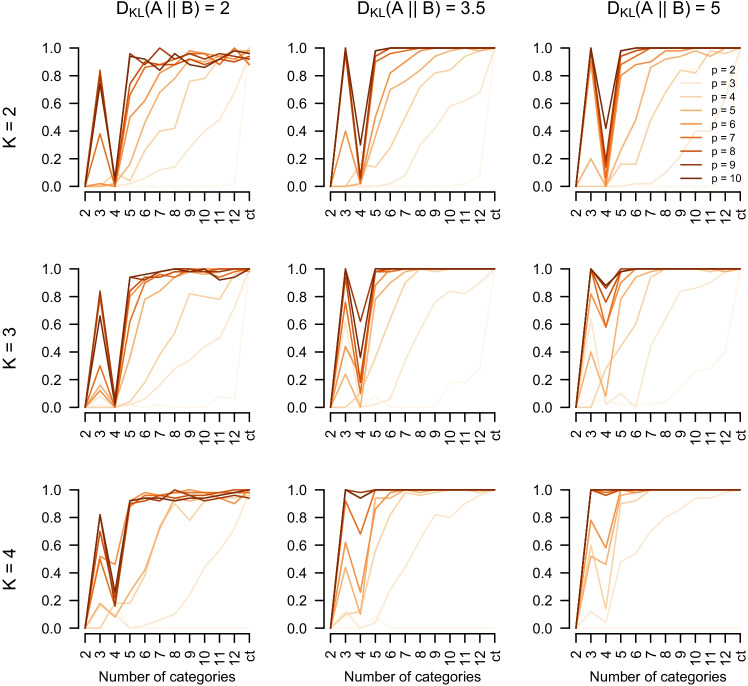
Probability of correctly estimating *K* (i.e., accuracy) as a function of *K* (*rows*), D_KL_ (*columns*), the number of ordinal categories (*x*-axes) and the number of dimensions *p* (*orange gradient*). The sample size is fixed to *N* = 10,000

We discuss the results of the constrained estimation in the main text and the results of the unconstrained estimation in Appendix B. Figure 3 displays the probability of recovering the correct number of components (i.e., accuracy) as a function of *K* (rows), D_KL_ (columns), *p* (orange gradient), and the number of ordinal categories (*x*-axis), fixed for *N* = 10,000. We discuss the results for *N* = 10,000 first because they are the most illustrative of the effects of the misspecification by ordinal scales.

**Fig. 4:**
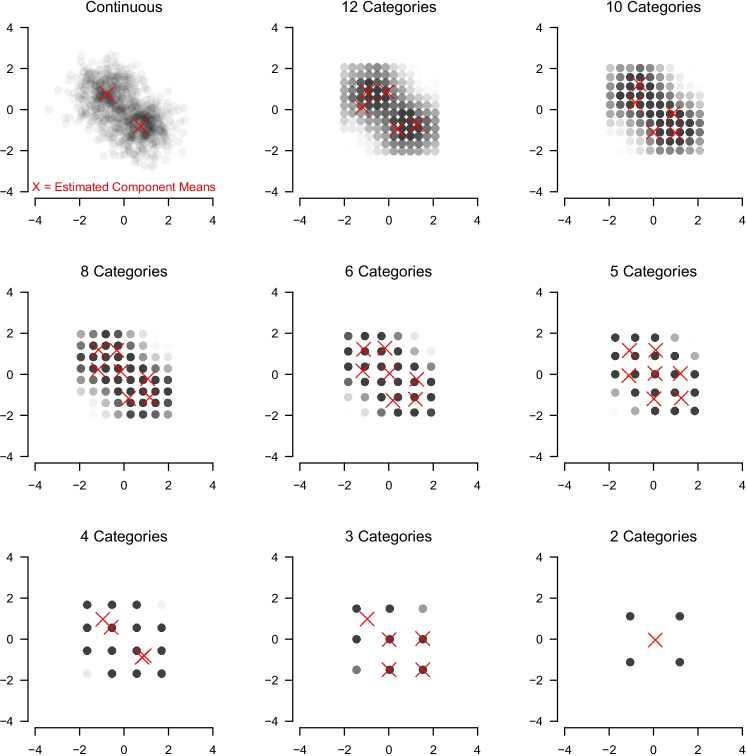
Bivariate Gaussian mixture (*top left*) is mapped to ordinal scales with 12, 10, 8, 6, 5, 4, 3 and 2 categories. The red Xs indicate the estimated component means

We first consider the bivariate case (*p* = 2). We see that, across the variation of D_KL_ and for  $K\ \in \{2,3 \}$, the performance is very low for small number of categories and tends to increase as the number of categories becomes larger. This increase is more rapid when *K* = 3 and D_KL_ are larger. The exceptions are the cases with *K* = 4. This is the only condition in which components varied in their covariances and hence estimation with diagonal covariances cannot recover these mixtures. This explains that accuracy is at zero for these cases (bottom row). In the bivariate case, we are able to visually inspect the data and the estimated component means: Fig. 4 displays the simulated data for *K* = 2, D_KL_ = 5 for different numbers of ordinal categories and the estimated component means (red Xs) in the first iteration of the simulation. If the data are continuous (top left panel), the component means are estimated correctly, as expected. However, when thresholding the continuous data into 12 ordinal categories, we estimate five components which are placed close to the true components means. When decreasing the number of categories to five, the over-estimation of *K* increases. From *c* = 4 on it decreases again and for *c* = 2 we underestimate with $\hat K = 1$. This shows, at least for the binary case, that observing ordinal categories strongly impacts GMM recovery.

**Fig. 5:**
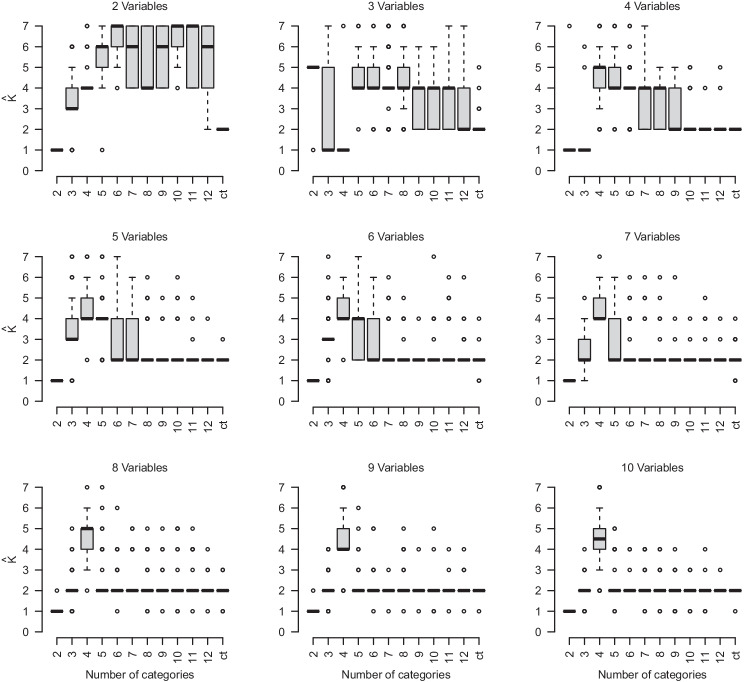
Box plots of the estimated number of components $\hat K$ for $p \in \{2,\dots , 10\}$ and fixed for *K* = 2, D_KL_ = 2, and *N* = 10,000

Considering *p* > 2 in Fig. 3, we observe a peculiar behavior in the accuracy that begins at zero at *c* = 2, increases at *c* = 3, *decreases* again at *c* = 4, and then increases to 1 as *c* increases further. The reason for this perhaps surprising behavior is that for small *c* the estimated *K* first increases from underestimation to overestimation, and then decreases again towards the correct *K*. This pattern is more pronounced for the conditions with more than two variables. Figure 5 displays this behavior in box plots of the estimated number of components $\hat K$, separate for $p \in \{2,\dots , 10\}$ and fixed for *K* = 2 and D_KL_ = 2.

We see that across all conditions with *p* > 2, the mean estimated $\hat K$ increases from *c* = 3 to *c* = 4 and then decreases again. This decrease after *c* = 4 is steeper for higher *p*. This non-monotone behavior of the location of the distribution of estimated $\hat K$s from underestimation (at *p* = 2) to increasing overestimation (for *p* ≤ 4) towards correct estimation (for *p* ≥ 4) explains the perhaps surprising non-monotone behavior of the accuracy measure as a function of *c* in Fig. 3. The underestimation at *c* = 2 is due to the fact that estimation for *K* > 1 fails due to singular covariance matrices of some of the components. Typically, the problem is that one component has a large variance and all other components have zero variance. This problem could be addressed by providing priors on the variances (Fraley et al., 2012; Scrucca et al., 2016). This behavior cannot be explained by the geometry of the true component means because we generate the configurations randomly and independently in each of the 100 simulation runs (see Appendix A).

Our best explanation is that two forces are at play: if the number of categories *c* is low the data looks roughly unimodal because the tails of the components collapse into grid points that have a probability mass similar to the grid points close to the true component means. In those cases, we expect that the components fall on the grid point masses. When going from *c* = 3 to *c* = 4, more such grid points are available, leading to higher *K*. When further increasing *c*, the true components become better separated because the grid points between the component means have smaller probability. This separation happens more quickly for larger *p*, which might explain why overestimation declines more quickly as a function of *c* for larger *p*.

**Fig. 6:**
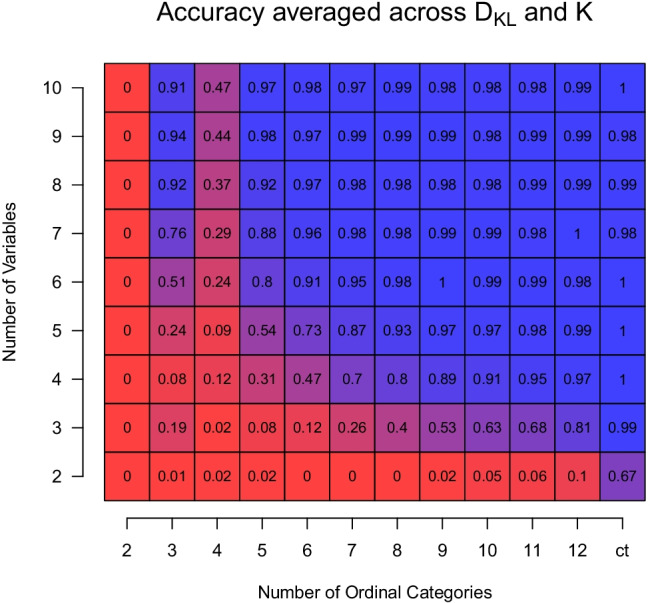
Accuracy averaged over *K* and D_KL_ and fixed for *N* = 10,000, conditional on the number of categories *c* (*x*-axis) and the number of variables *p* (*y*-axis)

From Fig. 3, we see that the number of true components *K* and the pairwise distance D_KL_ clearly impacts accuracy. However, we also see that accuracy is predominantly driven by the number of variables *p* and the number of ordinal categories *c*. To summarize our findings, we therefore average accuracy across *K* and D_KL_ and display it conditional on *p* and *c* in Fig. 6. We see that performance is extremely low if we have few variables and few ordinal categories. However, when having more than a few variables and more than a few ordinal categories, performance improves dramatically. For example, with only four variables and 12 ordinal categories we already achieve an average accuracy of 0.97. Or, if we have ten variables, *c* = 5 categories are enough to achieve high accuracy (0.97). Generally, average accuracy is high if *p* > 5 and *c* > 5. Thus, if we remain in those situations, GMMs can also be recovered with ordinal variables, if the sample size is high enough. The situation is a bit more complex for lower sample sizes, for which performance drops considerably when the components of the data-generating GMM are less well separated. However, even for *N* = 1000, performance is high for *p* > 5,*c* > 7 if the components are well separated (see below).

**Fig. 7:**
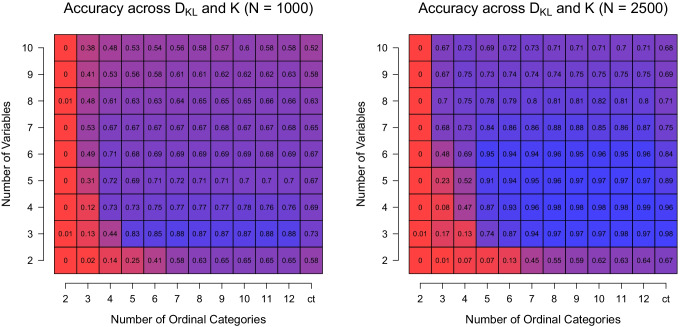
Accuracy averaged over *K* = 2 and D_KL_ = 2 and fixed for *N* = 1000 (*left*) and *N* = 2500 (*right*), conditional on the number of categories *c* (*x*-axis) and the number of variables *p* (*y*-axis)

So far, we only considered results for sample size *N* = 10,000. Figure 7 displays the same aggregate performance as Fig. 6 but for sample sizes  $N\ \in \{1000,\ 2500 \}$. For *N* = 1000 (left panel), we see the same pattern as in Fig. 6 showing the results for *N* = 10,000 when *c* and *p* are small: when the number of categories and dimensions are too small, the recovery performance is very low. When both increase, the performance increases. However, different to the setting with *N* = 10,000, we see that the performance decreases again when increasing *p* > 4. The reason for this behavior is that the number of parameters increases linearly with *p* (specifically, *K*(*p* + 1) + (*K* − 1)), which increases the weight of the penalty term of the BIC. We do not see this effect in the *N* = 10,000 because the likelihood part in the BIC has a much higher weight because we have more data. For *N* = 2500 (right panel), we see a similar pattern, except that the drop in performance as a function of *p* is much smaller since we have more data relative to the number of parameters.

In the above results, we only considered to what extent the number of components *K* can be correctly estimated. However, we did not explore yet how well the parameters of the GMM can be estimated when *K* has been estimated correctly. The additional parameters are the *K* − 1 mixing probabilities and the means and covariance matrices of each of the *K* components. Here, we report how well the means and covariances of each of the *K* components are estimated. To this end, we consider only those simulation results from the main text in which *K* has been estimated correctly. In each case, it is necessary to map each component in the estimated model to its corresponding component in the data-generating model. We do this by computing average errors for all *K*! possible mappings and choose the one with the smallest error.

**Fig. 8:**
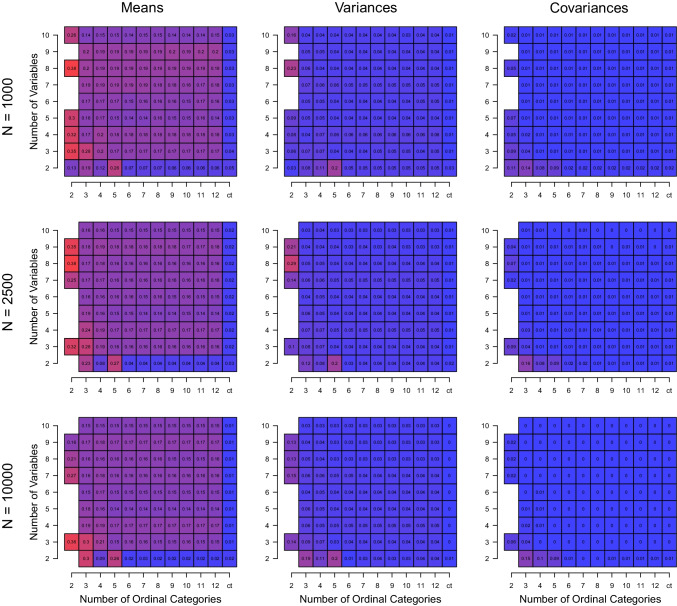
The mean absolute estimation error on the GMM parameter estimates for the *constrained* estimation considering only isomorphic covariance matrices, averaged over the variations of *K* and D_KL_, as a function of sample size *N* (*rows*), number of variables *p* (*y*-axes), and number of ordinal categories *c* (*x*-axes), separately for means, variances, and covariances (*columns*). Missing cells indicate that *K* was correctly estimated in none of the iterations of the simulation

To keep the number of presented results manageable, we take a similar approach to above and average over the variations in *K* and D_KL_. Figure 8 displays the mean squared difference between true and estimated parameters as a function of sample size (rows), number of variables *p* (*y*-axes) and number of ordinal categories *c* (*x*-axes), separately for means, variances, and covariances (columns), and rounded to two decimals. The missing cells indicate that the correct *K* was estimated in none of the simulation runs.

We observe average absolute errors around 0.15 for means across variations of the number of variables *p* and the number of ordinal categories *c*. These errors are relatively large considering that the true means vary roughly between − 0.5 and 1.5. Interestingly, this error does not seem to decrease when increasing the sample size. This shows that the error is due to the misspecification created by the ordinal mapping. In contrast, when the data are continuous, we see that the estimation error is extremely small across *N*. This shows that for the continuous case, once we correctly estimated *K*, we can also expect precise estimates of the mean vector. The estimation error in the variances and covariances is very low across all conditions, as one would expect, since we constrained all covariances to be isomorphic, which corresponds to the data-generating GMM.

The above results are of the estimation method in which we constrained the covariance matrices to be diagonal with equal variances. Except for the condition *K* = 4, *p* = 2, these models are correctly specified because also the data-generating GMMs have diagonal covariances with equal variances. When estimating covariance matrices unconstrained, we would expect a drop in performance, since the model has many more parameters while the sample size remains constant. In addition, we expect this effect to be larger for conditions with more dimensions *p* because the parameters grow quadratically with *p*. We report the results of estimating GMMs with unconstrained covariance matrices and verify those theoretical predictions in Appendix B. In addition to verifying these predictions, the main result of those additional results is that one requires a relatively large sample size in order to estimate a GMM with unconstrained covariance matrices because the number of parameters grow quadratically with the number of variables *p*.

An additional way to assess the performance of mixture/clustering methods is to consider to what extent cases are classified to the correct component or cluster. The classification performance depends on how well the mixture components are separated and how well the mixture distribution is being estimated. In the present paper, the focus is on the extent to which Gaussian mixture models can be recovered from data in various scenarios. We therefore chose performance measures capturing how well *K* is estimated and how well the parameters of component models are estimated once *K* has been estimated correctly. Classification performance captures estimation performance less directly, since it is a function of both estimation performance and the separation between components in the true mixture model. For this reason, and to keep the paper concise, we did not include classification performance.

## Discussion

In this paper, we explored to what extent the recovery of Gaussian mixture models (GMMs) is affected by observing ordinal instead of continuous data. Our focus was on recovery performance as a function of the number of ordinal categories, which we explored conditional on various characteristics of the data-generating GMM and sample size. We found that for large sample sizes (*N* = 10,000), the probability of correctly recovering *K* was high as long as both the number of categories and the number of dimensions were at least 6. For lower sample sizes (*N* = 1000 and *N* = 2500), this is only the case if the mixture components are well separated. However, when focusing on the estimation error on means and covariance matrices of the components, we found that the estimates were biased across all conditions with ordinal data.

As in any simulation study, we had to keep various parameters fixed, which limits the range of our conclusions. First, we assumed that the mixing probabilities are $\frac {1}{K}$. There is no reason why this should be the case in empirical data and the performance is likely to drop if the density is not the same across mixture components. Second, with the exception of the binary case with *K* = 4, we only used diagonal covariance matrices with equal variances. While we consider the KL-divergence to be the main driver of recovery performance, it is possible that different specifications of a GMM with fixed pairwise KL-divergence differ in how easy they are to recover (see Appendix C). It would therefore be interesting to study how different specifications of the GMM affect recovery performance. This would be especially relevant for applications where the focus is on differences in covariances across components, since we varied the separation between components only based on the mean vectors, yet we show in Appendix C that it matters for recovery whether a fixed KL-divergence is due to differences in means or covariances. Third, in the mapping from continuous to ordinal data, we used equidistant thresholds. Thresholds at unequal intervals are likely to make recovery more difficult. In fact, if taken to the extreme, thresholds could be chosen to create a multi-modal distribution out of a uni-modal Gaussian, which likely renders recovery impossible. These limitations suggest that our performance results represent the best-case scenario. Finally, we assumed a latent distribution in individuals that is mapped on ordinal categories through a set of thresholds. This ignores the fact that in practice responses are typically subject to measurement invariance / response styles (Paulhus, 1991). Such response styles are well studied for ordinal responses (e.g., Morren et al., 2011; Van Rosmalen et al., 2010; Tijmstra et al., 2018; Manisera & Zuccolotto, 2021) and could be modeled with an additional latent variable.

We showed that in some situations one can use standard GMM estimation with the EM algorithm and the BIC to correctly estimate the number of components. It is also possible to obtain reasonable estimates of the component parameters, but they are biased. In other situations, however, we showed that GMM recovery clearly fails. We also found peculiar results around *c* = 2,3,4 for *N* = 10,000 (e.g., Fig. 6), but did not observe the same qualitative behavior with smaller sample sizes. Finally, we showed that the parameter estimates of the component-distributions are biased, a problem that cannot be solved with larger sample sizes. These issues suggest the need for alternative modeling strategies.

One possible alternative could be to use models that assume a latent multivariate Gaussian that is thresholded into ordinal categories by a set of threshold parameters (e.g., Guo et al., 2015; Suggala et al., 2017; Feng & Ning, 2019). This idea can be extended to GMMs, in which the threshold parameters are allowed to differ across component models. Ranalli and Rocci (2016) and Lee et al., (2021) have put forward estimation procedures for such a thresholded latent GMM. These methods should outperform standard GMMs in our simulation setting, since we used the thresholded latent GMM as the data-generating mechanism. However, so far, no implementation of these methods is available in the mixture context. *Mplus* (Muthén and Muthén, 2017) allows one to threshold continuous variables into ordinal categories, however, in the mixture context the ordinal thresholds are class-specific, which leads to a large number of parameters.

Another alternative would be to consider mixtures of distributions that explicitly model variables as categorical. An example is the classic latent class model with polytomous responses, which can be estimated with the R-package *poLCA* (Linzer & Lewis, 2011). However, in this model, categorical variables are considered polytomous/nominal, which leads to a large number of parameters. In addition, this implementation does not allow one to introduce local dependencies to allow how groups differ in terms of interactions between variables across latent classes. This issue could be addressed by using mixtures of distributions that explicitly model the ordering of categorical (i.e., ordinal) variables, which reduces the number of parameters necessary to parameterize interactions considerably (Agresti, 2018). For example, Suggala et al., (2017) discusses a graphical model based on the consecutive (or adjacent-category) logit model. A mixture of the adjacent-category model is implemented in the commercial software *Latent GOLD* (Vermunt and Magidson, 2013), under the assumption that variables are independent within each component model. However, it is possible to introduce local dependencies to study how interactions between ordinal variables differ across components. We consider studying mixtures of multivariate distributions based on the adjacent-category model as a promising avenue for future research.

To summarize, we explored to what extent the recovery of Gaussian Mixture Models is impacted by observing ordinal instead of continuous data. We showed that the correct number of components can be estimated if the number of variables, the number of ordinal categories, and the sample size are high enough. However, a bias on the parameters of the component models also remains for high sample sizes. In light of these results, we discussed possible models that are better suited than the GMM to model heterogeneity in multivariate ordinal models. Specifically, extending latent Gaussian distributions or multivariate distributions explicitly for ordinal variables to the mixture context are promising directions for future work. We hope that our results help researchers to assess whether GMM estimation on their ordinal data is acceptable and that they motivate better methods to estimate mixtures multivariate ordinal models.

## Figures and Tables

**Fig. 9:**
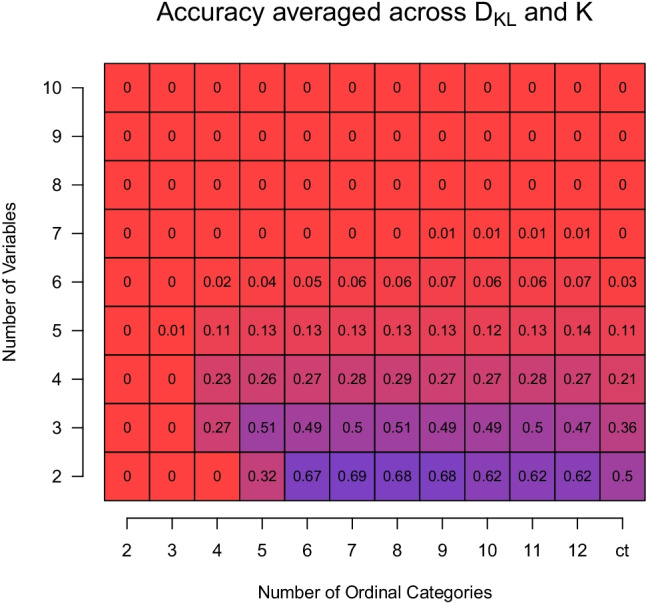
Accuracy averaged over *K* and D_KL_ and fixed for *N* = 1000, conditional on the number of categories *c* (*x*-axis) and the number of variables *p* (*y*-axis), for GMM estimation with unconstrained covariance matrices

**Fig. 10:**
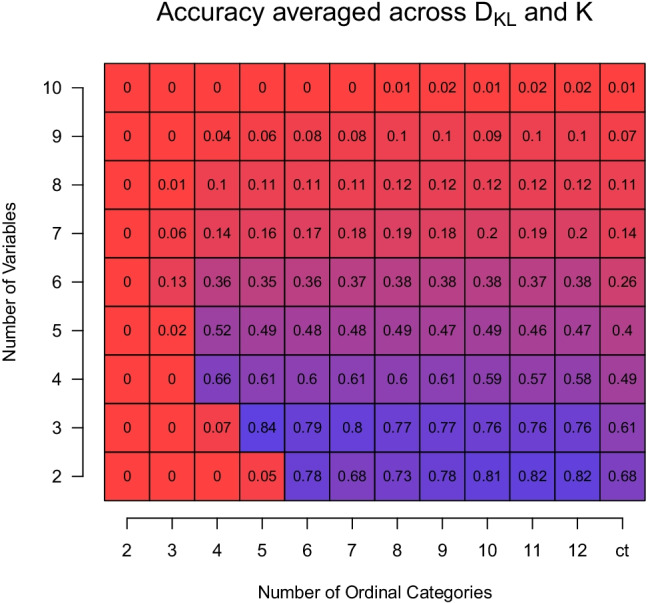
Accuracy averaged over *K* and D_KL_ and fixed for *N* = 2500, conditional on the number of categories *c* (*x*-axis) and the number of variables *p* (*y*-axis), for GMM estimation with unconstrained covariance matrices

**Fig. 11:**
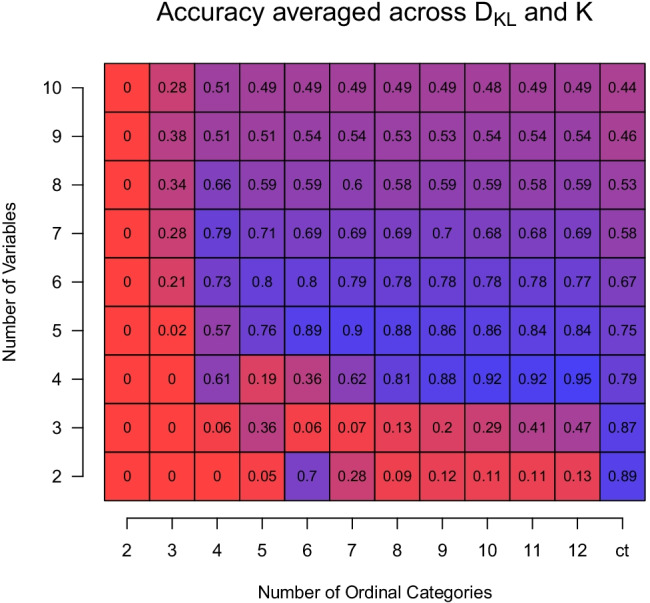
Accuracy averaged over *K* and D_KL_ and fixed for *N* = 10,000, conditional on the number of categories *c* (*x*-axis) and the number of variables *p* (*y*-axis), for GMM estimation with unconstrained covariance matrices

**Fig. 12:**
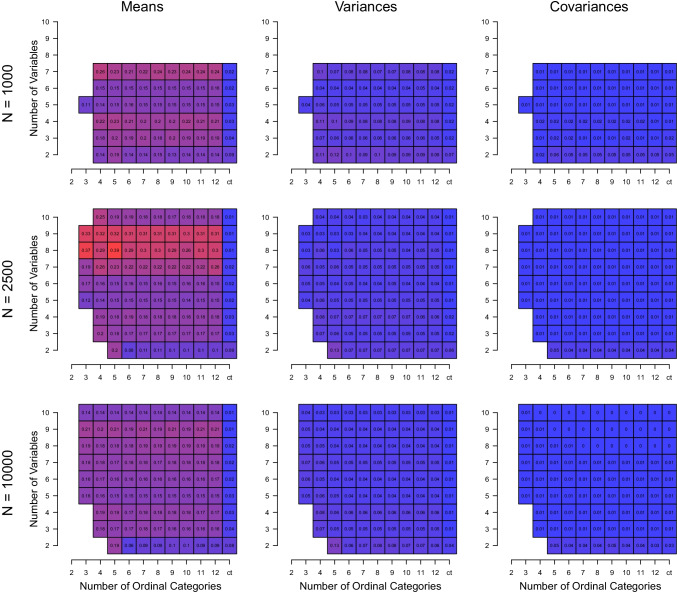
The mean absolute estimation error on the GMM parameter estimates for the *unconstrained* estimation, averaged over the variations of *K* and D_KL_, as a function of sample size *N* (rows), number of variables *p* (*y*-axes) and number of ordinal categories *c* (*x*-axes), separately for means, variances, and covariances (*columns*). Missing cells indicate that *K* was correctly estimated in none of the iterations of the simulation

**Fig. 13:**
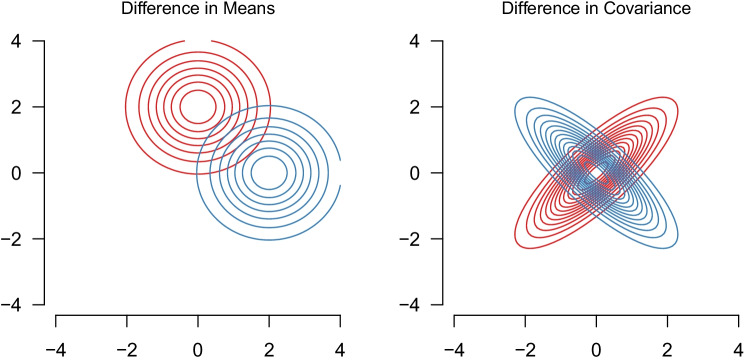
Contour plot of two GMMs with the same D_KL_ which differ in their mean vectors (*left*) or their covariance matrix (*right*)

## Appendix A: Specification of data-generating mixture components

For *p* = 2 and *K* = 2, 3, it is possible to put pairwise equidistant points into the plane. For *K* = 4, this is not possible and we therefore chose covariances that the pairwise D_KL_ is the same as for *K* = 2, 3. The exact values for all means, variances, and covariances are contained in the reproducibility archive attached to this paper. For dimensions *p* > 2, it is possible again to place *K* = 2, 3, 4 component means such that the distance between pairs of components is equal across pairs. In order to choose these means, we used numerical optimization, where the quantity minimized the squared difference between the current and the desired pairwise D_KL_ summed over the $\frac {K(K-1)}{2}$ pairwise distances. We initialized the means with draws from $\mathcal {U}(0, 1)$. The error tolerance was set to 0.01, which resulted in pairwise distances accurate to the second decimal.

## Appendix B: Results for GMM estimation with unconstrained covariance matrices

In the main text, we only reported the results for estimating GMMs with isomorphic covariance matrices. Here, we report the results for estimating GMMs in which covariances are unconstrained. To keep the results concise, we again focus on summary figures that average over the variations of *K* and D_KL_. Figures 9, 10, and 11 represent the aggregate results for the sample sizes *N* = 1000, 2500, and 10,000, respectively. The general pattern is the same as in the results in the main text,  except that the drop in performance as a function of *p* is more pronounced. This is because the number of parameters grows quadratically with *p*, when fully estimating all covariance matrices. Specifically, the number of parameters is *K*(*p* + *p*(*p* − 1)/2) + (*K* − 1). This is a relevant result for research trying to analyze individual covariances across components, because the present results show that such an analysis requires relatively high sample sizes.

Figure 12 displays the same results as Fig. 8 but for estimating the GMMs unconstrained. We see that more cells are missing, which corresponds to the fact that *K*-recovery is harder in this condition. We again see considerably large estimation errors across all conditions. The only difference to the results of constrained estimation is that for *N* = 2500 the errors are even larger for high *p*. For continuous data, we again see that estimation errors are extremely small across all conditions. Interestingly, the estimation errors in variances and covariances are very small across all conditions, despite the fact that they are estimated freely. However, this result may be contingent on using only isomorphic GMMs as data-generating mechanisms. It would be an interesting avenue for future work to investigate how well the number of components *K* and the component parameters can be recovered if GMMs vary (predominantly) in their covariance matrices.

## Appendix C: D_KL_ does not fully determine recovery performance

In the simulation study, we use the pairwise D_KL_ between mixture components as a way to vary the separation between components and thereby the difficulty of recovering the correct number of components *K*. While increasing D_KL_ clearly increases the performance in estimating *K* correctly, we found that it does not fully determine performance. That is, it is possible to specify two GMMs with fixed *p* which have the same D_KL_ pairwise, but differ in how difficult it is to recover *K*. In what follows, we study two examples, which show that it matters both whether one varies the mean vector or the covariance matrices, and, perhaps more surprisingly, it matters how differences in the mean vector are distributed across dimensions.

### C.1 Differences in means vs. covariances

We consider the two GMMs with *K* = 2 components and *p* = 2 dimensions. In the first GMM, the components differ in the mean vectors *μ*_1_ = [0, 2], *μ*_2_ = [2, 0], while the covariance matrices Σ_1_ = Σ_2_ = *I* are equal to the identity matrix. The second GMM has equal mean vectors *μ*_1_ = *μ*_2_ = [0, 0] but the covariances are 0.8165 and − 0.8165, respectively, while all variances are equal to 1. We chose the parameters such that the D_KL_ between the components is equal to 4 rounded to the 4th decimal. The contour plots of the two mixtures are shown in Fig. 13:

We simulated 100 datasets with *N* = 1000 from both GMMs and evaluated the accuracy in estimating *K*. The accuracy was 1.00 and 0.74 for the GMMs in the left and right panels, respectively. All errors were due to over-estimation of *K*. This shows that GMMs with differences in means are easier to recover than GMMs with differences in covariances, when keeping D_KL_ constant. This shows that D_KL_ does not fully determine how difficult it is to recover the correct *K*.

### C.2 Varying differences in means across dimensions

We consider two GMMs with *K* = 2 with equal covariance matrices and equal D_KL_ and study whether distributing differences in means across more or less dimensions impacts recovery performance. For two dimensions, it is easy to see that this distribution cannot impact recovery performance. For example, you could rotate the GMMs in the left panel of Fig. 13 with respect to the origin such that the mean difference is only in one dimension. Yet, clearly rotating the GMM should not impact the performance in recovering *K*. However, perhaps surprisingly, this is different in *p* > 2 dimensions. We demonstrate this by considering two GMMs with *p* = 10 dimensions and diagonal covariance matrices. In the first GMM, we specify a difference only in the first dimension by setting *μ*_1_ = [*𝜃*, 0,..., 0], *μ*_2_ = [0, 0,..., 0]. In the second GMM, we specify *μ*_1_ = [*γ*,*γ*,...,*γ*], *μ*_2_ = [0, 0,..., 0], where *𝜃* = 2.8281, *γ* = 0.8945 are chosen such that D_KL_ = 4 rounded to the 4th decimal for both GMMs.

We again simulated 100 datasets, this time with *N* = 10, 000, from both GMMs and evaluated the accuracy in estimating *K*. The accuracy was 0.50 and 1.00 for the GMMs in the left and right panels, respectively. The errors are all due to under-estimation. This shows that a GMM in which differences between components are distributed across dimensions is easier to recover than a GMM in which the difference is in one dimension, when keeping D_KL_ constant. Again, we see that D_KL_ does not fully determine how difficult it is to recover the correct *K*.
